# Immunotherapy in Oncogene-Addicted NSCLC: Evidence and Therapeutic Approaches

**DOI:** 10.3390/ijms26020583

**Published:** 2025-01-11

**Authors:** Lorenzo Foffano, Elisa Bertoli, Martina Bortolot, Sara Torresan, Elisa De Carlo, Brigida Stanzione, Alessandro Del Conte, Fabio Puglisi, Michele Spina, Alessandra Bearz

**Affiliations:** 1 Department of Medical Oncology, CRO di Aviano, National Cancer Institute, IRCCS, 33081 Aviano, Italy; elisa.bertoli@cro.it (E.B.); martina.bortolot@cro.it (M.B.); elisa.decarlo@cro.it (E.D.C.); brigida.stanzione@cro.it (B.S.); alessandro.delconte@cro.it (A.D.C.); fabio.puglisi@uniud.it (F.P.); mspina@cro.it (M.S.); alessandra.bearz@cro.it (A.B.); 2 Department of Medicine, University of Udine, 33100 Udine, Italy

**Keywords:** immunotherapy, oncogene-addicted non-small cell lung cancer, treatment resistance, new therapeutic strategies

## Abstract

Non-small cell lung cancer (NSCLC) remains a leading cause of cancer-related mortality worldwide. The discovery of specific driver mutations has revolutionized the treatment landscape of oncogene-addicted NSCLC through targeted therapies, significantly improving patient outcomes. However, immune checkpoint inhibitors (ICIs) have demonstrated limited effectiveness in this context. Emerging evidence, though, reveals significant heterogeneity among different driver mutation subgroups, suggesting that certain patient subsets may benefit from ICIs, particularly when combined with other therapeutic modalities. In this review, we comprehensively examine the current evidence on the efficacy of immunotherapy in oncogene-addicted NSCLC. By analyzing recent clinical trials and preclinical studies, along with an overview of mechanisms that may reduce immunotherapy efficacy, we explored potential strategies to address these challenges, to provide insights that could optimize immunotherapy approaches and integrate them effectively into the treatment algorithm for oncogene-addicted NSCLC.

## 1. Introduction

Lung cancer remains one of the most significant global health challenges, with approximately 1.8 million new diagnoses and 1.6 million deaths attributed to the disease annually. Despite substantial advancements in diagnostic and therapeutic strategies for both early- and advanced-stage disease, the 5-year survival rate remains alarmingly low, ranging from 4% to 17%, depending on the stage at diagnosis and geographical factors [1,2]. Non-small cell lung cancer (NSCLC), which accounts for around 80% of all lung cancer cases, has undergone a paradigm shift in both classification and treatment, driven by the identification of key oncogenic drivers [3]. The discovery of oncogenic mutations has led to the development of targeted therapies, particularly tyrosine kinase inhibitors (TKIs), which have transformed the clinical management of NSCLC, providing tailored approaches that significantly improve progression-free survival (PFS) and overall survival (OS) for this subset of patients.

Indeed, international guidelines from the International Association for the Study of Lung Cancer (IASLC) and the European Society for Medical Oncology (ESMO) recommend the assessment of a comprehensive Next Generation Sequencing (NGS) panel to identify predictive biomarkers, including epidermal growth factor receptor (*EGFR*) gene mutations, anaplastic lymphoma kinase (*ALK*) and *ROS* proto-oncogene-1 receptor tyrosine kinase (*ROS1*) rearrangements, *BRAF* V600 point mutations, c-mesenchymal-epithelial transition factor (*c-MET*) amplifications and exon 14 (*METex14*) skipping mutations, neurotrophin tyrosine kinase (*NTRK*) gene fusions, rearranged during transfection (*RET)* rearrangements, and Kirsten rat sarcoma proto-oncogene (*KRAS)* G12C and human epidermal growth factor receptor 2 (*ERBB2*) mutations, along with Programmed Death-Ligand-1 (PD-L1) evaluation [4,5,6]. The mutation of receptors or kinases encoded by driver oncogenes often leads to the constitutive activation of signaling pathways that promote cell survival.

While targeted therapies have shown remarkable efficacy, inevitably disease progression occurs due to the development of resistance mechanisms. In parallel, immune checkpoint inhibitors (ICIs) targeting PD-1/PD-L1 and cytotoxic T-lymphocyte associated protein 4 (CTLA-4) have emerged as game-changing therapies in NSCLC therapy, firstly in the advanced setting of the disease, becoming the standard first-line therapies as a monotherapy or in combination with chemotherapy in non-oncogene-addicted NSCLC, and more recently in the early setting [7,8]. Nevertheless, patients with driver mutations generally demonstrate limited benefit from ICIs therapy, as evidenced by early clinical trials that subsequently led to their exclusion from many later studies [9,10]. Consequently, current evidence is primarily derived from retrospective analyses, resulting in a relatively narrow proof base. Notably, preclinical and clinical studies have suggested significant heterogeneity both within and between different driver mutation subgroups, showing clinical and biological variations that may influence responsiveness to immunotherapy.

Given this high unmet clinical need, research has intensified to understand the complex interplay between oncogenic drivers and the tumor immune microenvironment (TME), aiming to identify new strategies to improve the efficacy of immunotherapy in oncogene-addicted NSCLC.

The aim of this review is to provide a comprehensive overview of the current evidence on the role of ICIs in oncogene-addicted NSCLC, focusing on mechanisms of resistance and exploring whether, and under what conditions, patients with oncogene-driven NSCLC could benefit from ICIs, either as a monotherapy or in combination with chemotherapy or targeted therapies.

## 2. Intrinsic Mechanisms of Resistance to ICIs in Oncogene-Addicted NSCLC

Extensive preclinical and clinical investigations have sought to elucidate the molecular and histological factors underlying the variable responses to immunotherapy in oncogene-driven tumors. Two primary profiles emerge as critical in modulating responses: the tumor-intrinsic immunogenicity and the characteristics of the surrounding TME.

The oncogenic signaling pathways associated with specific driver mutations can either enhance or suppress immune responses through various mechanisms. A critical, though still debated, factor is the degree of PD-L1 expression associated with different oncogenic drivers. A pooled analysis of 15 clinical trials demonstrated a significant reduction in PD-L1 expression in NSCLC patients harboring *EGFR* mutations (OR 1.79, *p* = 0.02) [11]. Preclinical data suggest that the constitutive activation of the *EGFR* pathway can lead to the inactivation of Interferon Regulatory Factor 1 (*IRF1*), thereby suppressing interferon-γ-induced PD-L1 expression [12], and a meta-analysis of 26 studies further corroborated an inverse correlation between *EGFR* mutations and PD-L1 expression (OR 0.64, *p* = 0.014) [13]. In contrast, the same analysis demonstrated that *KRAS* mutations were positively correlated with PD-L1 expression (OR 1.45, *p* = 0.001) [13], consistent with preclinical findings indicating that *KRAS* signaling stabilizes PD-L1 (CD274) mRNA, thereby upregulating its expression [14]. Furthermore, an analysis of 1586 NSCLC patients with paired PD-L1 testing and next-generation sequencing revealed that *KRAS* mutations, unlike *EGFR* mutations, were significantly associated with high PD-L1 expression, a trend also observed in tumors harboring tumor protein P53 (*TP53*) and *MET* mutations [15].

Beyond PD-L1 expression, tumor mutational burden (TMB) plays a pivotal role in determining the efficacy of immune checkpoint inhibitors. TMB is defined as the total number of mutations per megabase (mut/Mb) in the coding regions of the genome and is conventionally regarded as a marker of immunotherapy responsiveness. By serving as a surrogate for the tumor’s neoantigen load, TMB reflects the potential number of novel peptides presented to T cells, thereby contributing to an immunogenic activation. In the context of NSCLC, higher TMB levels often correlate with robust immune cell infiltration and a heightened T cell–mediated inflammatory response, resulting in increased sensitivity to PD-1/PD-L1 blockade—an effect that can manifest even in tumors with otherwise low PD-L1 expression [16].

Despite its clinical utility, TMB remains an imperfect biomarker. Technical and methodological challenges, such as variability in sequencing platforms and differences in bioinformatic pipelines, can hinder reproducibility across laboratories [17]. Moreover, only a fraction of identified mutations give rise to true neoantigens capable of eliciting a sustained immune response, further limiting the prognostic and predictive reliability of TMB [18]. Tumors with *EGFR*, Human Epidermal Growth Factor Receptor 2 (*HER2*), and *ALK* alterations consistently exhibit lower TMB, suggesting a reduced neoantigen load that may contribute to the diminished immunogenicity [19]. In contrast, *KRAS*-mutant tumors exhibit significantly higher TMB, partly attributable to their strong association with smoking-related mutagenesis [20,21]. Moreover, emerging preclinical data suggest that specific *KRAS*, *BRAF*, and phosphatidylinositol-4,5-bisphosphate 3-kinase catalytic subunit alpha (*PIK3CA*) mutations may generate novel neoantigenic epitopes, potentially increasing mutational heterogeneity and thereby enhancing responsiveness to immunotherapy [22].

The tumor microenvironment also plays a pivotal role in modulating the immune response to ICIs, considering that tumors characterized by a less inflamed TME are generally less responsive to immunotherapy. Both *KRAS* and *EGFR* mutations are known to drive the production of pro-inflammatory cytokines that promote an immunosuppressive microenvironment. For example, *KRAS* mutations induce the secretion of Interleukin-1 alpha (IL-1α), Interleukin-1 beta (IL-1β), and Interleukin-6 (IL-6), while *EGFR* mutations drive the production of C-C Motif Chemokine Ligand 18 (CCL18), C-X-C Motif Chemokine Ligand 1 (CXCL1), and IL-1β, which collectively contribute to immune evasion [23,24,25]. Additionally, *EGFR*-mutant tumors exhibit reduced CD8+ T cell infiltration and lower T cell receptor clonality, further limiting their immunogenic potential. Notably, in the case of *KRAS* mutations, the strong association with smoking status and the resulting high mutational burden enhance neoantigen load, partially counteracting their immunosuppressive effects and increasing responsiveness to ICIs. Compounding this, defects in antigen presentation—such as the loss of heterozygosity (LOH) at the Human Leukocyte Antigen (HLA) Class I locus—are increasingly recognized as a mechanism of immune evasion in oncogene-addicted tumors [26], impairing T cell activation/differentiation and thus limiting the efficacy of immune-based therapies.

## 3. Efficacy of Immunotherapy in NSCLC with Oncogene Drivers

### 3.1. EGFR

*EGFR* mutations represent the most prevalent actionable genetic alterations in NSCLC, occurring in approximately 15–20% of patients with adenocarcinoma histology, particularly among non-smokers and East Asian populations. The in-frame deletion in exon 19 and a point mutation at codon 858 in exon 21 (L858R) account for up to 90% of all *EGFR* mutations [27,28]. For *EGFR*-mutant NSCLC, *EGFR* TKIs are the standard of care, as they have significantly improved PFS and OS compared to chemotherapy [29,30,31,32].

Preclinical data suggest that *EGFR* activation induces PD-L1 expression through mechanisms such as the phosphorylated extracellular signal-regulated kinases 1/2/ phosphorylated c-Jun (p-ERK1/2/p-c-Jun) signaling pathway [33] and inhibition of Nuclear Factor kappa-light-chain-enhancer of activated B cells (NF-κB) [34], leading to T cell apoptosis and the suppression of the immune response. This finding raised the hypothesis that *EGFR*-TKIs might indirectly enhance antitumor immunity by downregulating PD-L1 expression. In fact, a pooled analysis of 15 studies suggested that *EGFR*-mutant NSCLCs had decreased PD-L1 expression [11]. Moreover, *EGFR* mutations were associated with decreased TMB compared with wild-type *EGFR* [35], providing a biological rationale and a potential explanation for the limited responsiveness of this subgroup to immunotherapy.

Furthermore, early clinical trials evaluating ICI monotherapy showed limited efficacy in patients with *EGFR* mutations [9,10,36,37]. Moreover, numerous prospective studies, such as the phase II/III KEYNOTE-10 trial with pembrolizumab monotherapy [38], the phase I CheckMate012 trial with nivolumab [39], the phase III OAK [40] and the phase II BIRCH trials with atezolizumab [41], and the ATLANTIC trial with durvalumab [42], have consistently demonstrated a limited or absent benefit of immunotherapy compared with chemotherapy in previously treated *EGFR*-mutated NSCLC, together with several meta-analyses and retrospective studies [9,43,44] (Table 1). Specifically, data from the IMMUNOTARGET registry, which included 551 patients with oncogene-driver mutations treated with ICIs, showed that among the 125 patients harboring an *EGFR* mutation, the median PFS was 2.1 months (ranging from 1.4 months for T790M mutations to 2.5 months for exon 21 alterations) with a median OS of 10 months, further confirming the limited benefit of ICI monotherapy in this subgroup [43].

Similarly, a recent meta-analysis reported a pooled ORR of 6% in clinical trials and 8% in retrospective studies, with a median PFS of 2.77 months and a median OS of 9.98 months [45].

Given the limited efficacy of ICI monotherapy in *EGFR*-mutated NSCLC, several studies have evaluated the potential benefit of a combination with chemotherapy. Regarding the first-line setting, in the CheckMate 012 trial the combination of nivolumab and chemotherapy demonstrated limited efficacy as a first-line treatment in the *EGFR*-mutated subgroup compared to those with wild-type *EGFR* (median PFS of 4.8 versus 7.5 months, respectively) (Table 1) [46]. For TKI-resistant patients, along with some retrospective analysis [47,48], a phase II study examining the combination of toripalimab (an anti-PD1) with chemotherapy reported an overall response rate (ORR) of 50% and a median PFS of 7 months [49], whereas the CheckMate 722 found no significant PFS improvement with nivolumab and chemotherapy over chemotherapy alone (median PFS 5.6 vs. 5.4 months, HR 0.75, *p* = 0.0528) [50] (Table 1). Consistent with these observations, the most recent phase III data from the KEYNOTE-789 trial assessed the efficacy and safety of adding pembrolizumab to chemotherapy in TKI-resistant, *EGFR*-mutant metastatic non squamous NSCLC [51] (Table 1). Pembrolizumab plus chemotherapy did not yield significant improvements in PFS or OS over chemotherapy alone, reaffirming the minimal benefit offered by this strategy without the prior identification of predictive biomarkers for this patient subset.

Among the combination strategies explored in this setting, the addition of ICIs to *EGFR* TKIs has shown encouraging efficacy in early-phase trials [52,53,54,55,56] (Table 1). The phase I TATTON trial reported an ORR of 43% with osimertinib and durvalumab [52], while other studies demonstrated ORRs of 75% and 41.7% with atezolizumab plus erlotinib [53] and pembrolizumab plus erlotinib [54], respectively. Despite these promising response rates, each of these combinations was associated with significant toxicity, ultimately leading to the discontinuation of this treatment approach. In the phase Ib TATTON trial, 38% of patients developed interstitial lung disease (ILD) [52], while the atezolizumab–erlotinib combination led to high rates of hepatotoxicity [53], as similarly observed in the KEYNOTE 021 study [54], prompting the early termination of patient recruitment in the phase III CAURAL trial [56]. Notably, an increased incidence of treatment-related toxicity rates has also been observed not only in combination regimens but also in sequential treatment approaches. An analysis from the FDA Adverse Event Reporting System (FAERS), involving 20,516 patients, identified a higher rate of *EGFR* TKI-associated interstitial lung disease (ILD) in NSCLC patients who had received prior nivolumab treatment [57], aligning with findings from other clinical and preclinical studies [58,59,60]. As a result, while combination strategies have largely been abandoned due to severe immune-related adverse events, even sequential therapy may require cautious application with rigorous clinical, laboratory, and radiological monitoring.

### 3.2. EGFR Ex20Ins

Exon 20 insertions (Ex20Ins) account for 5–7% of EGFR-mutated NSCLC and are a major mechanism of intrinsic resistance to EGFR TKIs, representing a distinct NSCLC subgroup. In this context, the bi-specific antibody amivantamab has received FDA and EMA approval while the worldwide approval for mobocertinib has been withdrawn [61,62]. While data on the efficacy of ICIs in patients with EGFR Ex20Ins mutations are limited and most studies report modest outcomes, retrospective analyses have demonstrated improved ORR and PFS in this subgroup of patients treated with immunotherapy compared to those with classical EGFR mutations [63,64,65,66,67,68]. A recent retrospective analysis of 51 patients harboring an Ex20Ins alteration showed a significant improvement in PFS with first-line chemotherapy combined with ICIs compared to chemotherapy alone (10.3 vs. 6.3 months, *p* = 0.013) [65]. Similarly, a real-world analysis of 72 patients with Ex20Ins confirmed a significantly longer median PFS for those receiving immunotherapy compared to chemotherapy alone (10.7 vs. 4.6 months, *p* < 0.001), along with a trend toward an increased ORR (50% vs. 21.9%, *p* = 0.096) [66]. In contrast, an analysis from the Advanced NSCLC Flatiron Registry reported a median real-world time to next therapy (rwTTNT) of 3.7 months for the Ex20Ins group compared to 5.8 months for the wild-type group, with an HR of 1.58 indicating a significant risk for shorter time to next-line therapy; no significant differences emerged in the median real-world OS (10.9 vs. 11.3 months, respectively) [67].

A more recent retrospective analysis conducted by IASLC and the American Society of Clinical Oncology (ASCO), encompassing the largest dataset to date on patients with EGFR Ex20Ins mutations, included outcomes for 357 patients. Focusing on stage IV patients, those who received ICI treatment showed a significantly longer median OS compared to those who did not (median OS 29.1 vs. 14.7 months, *p* = 0.01) and a similar benefit was observed for patients treated with ICI plus chemotherapy compared to chemotherapy alone (median OS 29.1 vs. 16.5 months, *p* = 0.05) [68].

While most of the data were collected before the availability of amivantamab and should therefore be cautiously interpreted in current clinical practice, these findings confirm the view that patients harboring an EGFR Ex20Ins mutation represent a distinct subtype from classical EGFR mutations, showing resistance to standard TKIs while retaining a degree of sensitivity to immunotherapy. Further prospective studies, alongside predictive biomarkers, are needed to validate this hypothesis and potentially expand the therapeutic options for this subgroup.

### 3.3. ERBB2

*ERBB2* alterations occur in approximately 3% of non-squamous NSCLCs, including amplifications, overexpression, and, most notably, exon 20 insertions, which account for 80% of all cases [69]. Although chemotherapy and HER2-targeted therapies have shown inconsistent results in patients with HER2-positive NSCLC [43,70,71], the phase II DESTINY-Lung01 and DESTINY-Lung02 trials led to the approval of trastuzumab deruxtecan (T-DXd) in this subgroup, demonstrating significant clinical benefit [72,73]. It is likely *EGFR*-mutant NSCLC and HER2-mutant tumors have low PD-L1 expression and TMB as they share a similar epidemiological profile (young non-smoking patients, predominantly women). In a retrospective analysis of 122 patients [74], PD-L1 expression was <1% in 77% of cases, whereas only 13% showed PD-L1 levels > 50%, in line with other studies confirming a minimal proportion of patients with PD-L1 > 50% [75]. Consequently, minimal benefits have been observed from immunotherapy in these patients [43,76]. In the retrospective, multicenter IMAD2 study, which enrolled 23 patients harboring an *ERBB2* mutation, the median PFS was 2.2 months, with a 6-month PFS rate of 33.3% and a 12-month PFS rate of 22.9%, while the median OS was 20.4 months [76]. Similar findings emerged from other retrospective studies, with median PFS values ranging from 1.9 to 3.4 months [77].

A recent real-world retrospective analysis by Garassino et al. evaluated outcomes in 91 patients with an activating *ERBB2* mutation, including 65 with an *ERBB2* exon 20 insertion, who received first-line immunotherapy [78]. Compared to other driver alterations such as *BRAF* V600E, *METex14* mutation, and *MET* amplification, the median real-world time on treatment (rwTOT) for mono-immunotherapy was about 3 months shorter than those observed in the other cohorts and consistent with findings from the IMMUNOTARGET registry (median PFS of 2.1 months) [43]. In contrast, the combination of ICI and chemotherapy showed a rwTOT not significantly different from other cohorts, suggesting no detrimental effect, as indicated by an additional real-world retrospective analysis [79]. Similarly, a single-arm meta-analysis by Zhang et al., which examined 12 studies enrolling 260 patients, demonstrated a median PFS of 5.6 months with single-agent immunotherapy compared to 7.10 months with the combination of chemo-immunotherapy, confirming the potential benefit of this approach [80].

Interestingly, a case series (*n* = 5) reported clinical responses with first-line chemo-immunotherapy, yielding a median PFS of 8 months in this subgroup [81]. However, findings from the real-world POLISH study did not demonstrate an improved median PFS with chemo-immunotherapy compared to chemotherapy alone (*p* > 0.05), similar to observations by Zheng et al. [82,83].

Since, like for other targetable mutations, most available data derive from retrospective evidence, the role of immunotherapy in patients with *ERBB2* mutations remains under debate. Compared to immunotherapy alone, the chemo-immunotherapy combination has shown some promising efficacy signals with an acceptable safety profile, yet more robust prospective data are necessary to establish it as a viable alternative to chemotherapy alone. Encouraging insights may emerge from ongoing trials investigating immunotherapy in combination with antibody–drug conjugates (ADCs). Preclinical data suggest that ADCs’ pharmacodynamics might enhance T cell activity and increase MHC Class I expression on tumor cells, indicating a potential synergistic effect when combined with immunotherapy [84]. In this context, phase I/II trials assessing combinations of T-DXd or other ADCs with immunotherapy could provide valuable updates to the therapeutic algorithm for this patient subgroup [85,86,87].

### 3.4. ALK and ROS1 Rearrangement

The kinase domains of ALK and ROS1 exhibit approximately 70% homology, accounting for the overlapping clinical characteristics observed in ALK-rearranged and ROS1-rearranged NSCLC, as well as their shared susceptibility to ALK/ROS1 inhibitors, including crizotinib and lorlatinib [88]. Despite the relatively higher PD-L1 expression seen in these molecular subtypes compared to EGFR- and HER2-mutated tumors, their consistently low TMB is likely a key factor underlying the limited efficacy of ICIs in this population [20]. A subgroup analysis from the phase II ATLANTIC trial revealed significantly reduced activity of durvalumab in ALK-rearranged NSCLC compared to ALK wild-type [42]. These results were further corroborated by a recently published metanalysis, which reported a pooled ORR of 0% in patients with ALK rearrangements receiving ICI monotherapy in three clinical trials and of 3% in eight retrospective studies [45]. Considering these limited outcomes, the potential of combining ICIs with ALK/ROS1 TKIs in pretreated patients has been investigated. Nevertheless, various phase I/II studies, including those for the combinations of nivolumab with crizotinib [89], avelumab with lorlatinib [90], nivolumab with ceritinib [91], and alectinib with atezolizumab [92], were burdened by increased adverse event rates, particularly hepatotoxicity, frequently leading to trial discontinuation for treatment-related fatalities, without adding substantial outcome advantage in this subgroup of patients (Table 1). Interestingly, similar to the outcomes observed in EGFR-mutated NSCLC, the IMpower150 trial reported encouraging signals with the combination of chemotherapy, immunotherapy, and bevacizumab in ALK-rearranged tumors [93].

ROS1-positive NSCLC cells generally exhibit low PD-L1 expression and are associated with a low mutational load [94]. Despite the paucity of available data in the literature making it challenging to draw definitive conclusions, in the IMMUNOTARGET registry, ROS1-altered NSCLC exhibited an ORR of 17%, with 42.9% of patients classified as rapid progressors (within 2 months) [43]. In the LC-SCRUM-Japan study, among the 15 identified patients harboring an *ROS1* mutation, despite 53% having high PD-L1 expression (>50%), no response to ICI was detected [95]. Similarly, in the largest multi-institutional study of ROS1-rearranged NSCLC treated with immunotherapy, the ICI monotherapy subgroup demonstrated a median TTD of 2.1 months and an ORR of 13% [94]. Interestingly, more encouraging results were observed with the combination of chemo-ICI, showing a median TTD of 10 months and an ORR of 83% [94], suggesting the potential role of chemo-ICI combination therapy in this patient subgroup. In an effort to identify more robust predictive biomarkers, the same analysis undertook a comprehensive evaluation of the correlation between PD-L1 expression, TMB, and the therapeutic outcomes observed in patients treated with either ICI alone or chemo-ICI, yet ultimately failed to detect any statistically significant associations between these biomarkers and treatment response [94].

### 3.5. RET Rearrangement

*RET* fusions, with *KIF5B* being the most frequent fusion partner, represent an actionable target in NSCLC, although their incidence remains low, occurring in only 1–2% of cases [96]. The treatment landscape for this patient subgroup has been transformed by the results of the phase II LIBRETTO [97] and ARROW trials [98], which led to the approval of *RET*-specific TKIs such as selpercatinib and pralsetinib in the metastatic setting. Similar to *ALK* and *ROS1* fusions, *RET*-rearranged tumors exhibit variable PD-L1 expression; however, they are among the most immunologically ‘cold’ lung cancers, characterized by a median TMB of 1.75 mutations/MB, low neoantigen production, and intrinsic resistance to immunostimulatory therapies [99], seemingly independent of PD-L1 expression alone [100]. Although the low incidence of *RET* fusions has limited the available data to largely retrospective analyses, *RET*-rearranged tumors have consistently demonstrated a poor response to ICIs, with objective response rates below 10% and a PFS of 2.1 months, as reported in the IMMUNOTARGET trial [43,99]. In a retrospective analysis conducted by Hegde et al., which included 70 patients with solid tumors harboring *RET* alterations, non-ICI therapy was associated with a significantly reduced risk of treatment discontinuation compared to ICI therapy in the overall cohort (HR = 0.31; 95% CI 0.16–0.62; *p* = 0.000834) as well as among patients with *RET* point mutations (HR = 0.13; 95% CI 0.04–0.45; *p* = 0.00134). Among patients with *RET* fusions, non-ICI therapy also demonstrated a trend toward a reduced risk of treatment discontinuation (HR = 0.59; 95% CI 0.25–1.4; *p* = 0.24), supporting the prioritization of non-ICI over ICI therapy in patients with RET-positive tumors [101]. Similarly, a recent analysis by Yan et al. [100], which evaluated 38 patients with *RET* fusions treated with ICI-based regimens, reported a median PFS of 5 months, a median OS of 18 months, and an ORR of 26.3%, independent of PD-L1 expression and treatment line, thereby confirming the limited efficacy of ICI in this patient subgroup [97]. Contrary to these findings, a real-world study by Guisier reported a more favorable outcome with ICI monotherapy in *RET*-rearranged NSCLC, showing an ORR of 37.5% and a median PFS of 7.6 months [76], suggesting a potential variability in response within this population.

### 3.6. KRAS Mutations

*KRAS* mutations are the most prevalent genetic alterations observed in NSCLC, particularly affecting codons 12 and 13, with a prevalence estimated at 20–30% [102,103]. The CodeBreaK100 and CodeBreaK200 trials and the KRYSTAL-1 trial demonstrated the clinical efficacy of the *KRAS* G12C-specific inhibitors sotorasib and adagrasib, respectively, in pretreated patients harboring *KRAS* G12C mutations [104,105,106]. Notably, unlike other common driver mutations, *KRAS* G12C alterations are strongly correlated with a history of smoking. This association not only contributes to a higher TMB but also to elevated PD-L1 expression levels, which may explain the relatively enhanced response to immunotherapy in this patient subset when compared to other oncogene-driven tumors [20,21]. The relationship between KRAS mutations, particularly *KRAS* G12C, and response to immunotherapy has been a subject of growing interest [107,108]. The CheckMate 057 trial provided early insights into this association, demonstrating a marked improvement in OS with nivolumab compared to docetaxel in pretreated advanced NSCLC patients with *KRAS* mutations, yielding a HR of 0.52 [109] (Table 1). This observation was reinforced by subsequent FDA pooled analyses which revealed a comparable median ORR (37% vs. 33%, respectively) and OS (16.2 vs. 16.4 months, respectively) between *KRAS*-mutant and *KRAS* wild-type populations, further validating the potential for immunotherapy in this context [110]. In alignment with these findings, data from the IMMUNOTARGET registry highlighted that among patients with oncogene-driven NSCLC treated with ICIs, those harboring *KRAS* mutations exhibited the most favorable outcomes, with a response rate of 26% [43]. This was notably higher than the average response rate of 12.7% observed in patients with other targetable mutations, underscoring the distinct immunological behavior of KRAS-mutant tumors and their potential susceptibility to immunotherapeutic strategies.

However, it is increasingly evident that *KRAS*-mutated tumors are not a homogenous entity [111] but rather consist of distinct biological subgroups, with consequent important implications for treatment. Skoulidis et al. proposed a molecular classification of *KRAS*-mutated NSCLC into three distinct subgroups: the KL subtype (co-occurring Serine/Threonine Kinase 11 (STK11) and Kelch-like ECH-associated protein 1 (KEAP1) mutations), the KP subtype (associated with TP53 mutations), and the KC subtype (characterized by CDKN2A/B alterations and reduced TTF-1 expression) [112]. These subgroups exhibit differential responses to ICIs, with the KP subtype demonstrating higher levels of tumor-infiltrating lymphocytes (TILs) and increased PD-L1 expression, leading to more favorable outcomes with immunotherapy. Conversely, the KL and KC subtypes are generally considered “cold” tumors, characterized by lower response rates and poorer OS due to their reduced immunogenicity [112,113].

Given the promising results of KRAS-targeted therapies, there is are ongoing explorations of combination strategies involving ICIs. The phase Ib CodeBreak 100/101 trial investigated the efficacy of combining sotorasib with immunotherapy, where 58 patients were randomized to either a lead-in dose of sotorasib followed by atezolizumab or pembrolizumab, or a concurrent combination of sotorasib with either of the ICIs [114] (Table 1). While the study showed a median ORR of 29% and a median duration of response of 17.9 months, the combination therapy was also associated with a significant incidence of grade 3 or higher adverse events, particularly hepatotoxicity [114], highlighting the need for the cautious optimization of combination strategies to balance efficacy and safety.

In terms of current treatment guidelines, while selective KRAS G12C inhibitors continue to be investigated, the standard of care for advanced-stage KRAS-mutant NSCLC in the first line setting includes anti-PD-(L)1 monotherapy for patients with a PD-L1 tumor proportion score (TPS) ≥ 50% or chemoimmunotherapy, with or without bevacizumab, regardless of PD-L1 expression [4]. These treatment options are currently prioritized as first-line therapies, given their demonstrated efficacy in this challenging subset of NSCLCs, as recently confirmed by a meta-analysis of 86 studies, both prospective and retrospective, which reported a pooled ORR of 23% in clinical trials and 28% in prospective studies [45].

### 3.7. BRAF Mutations

*BRAF* mutations are detected in approximately 2% of NSCLC. *BRAF*-mutant NSCLC is a heterogeneous disease, as three distinct functional classes have been identified, with different biological characteristics and varying degrees of *RAF* kinase activation [115]. Class I mutations, which include all V600 variants and account for approximately 50% of cases, result in a strong constitutive activation of the *BRAF* kinase and therefore of the *MAPK* pathway; in this subgroup, a combination therapy with *BRAF* and *MEK* inhibitors improved survival and is recommend as first-line treatment. In contrast, Class II encompasses mutations and fusions that exhibit intermediate to high levels of kinase activity and Class III mutations are characterized by a minimal or absent enzymatic activity. For those with non-V600 *BRAF* mutations the benefit of targeted therapies remains debated and unclear, with chemotherapy still being the preferred treatment [116].

Compared to other actionable oncogenic alterations (AGAs), *BRAF* mutations seem to be associated with a susceptibility to immunotherapy analogous to the one highlighted in the wild-type population [76]. In the Zhang et al. study, the OS for patients with *BRAF*-mutant and BRAF-wild-type NSCLC treated with ICIs was 10 months and 11 months, respectively (*p* = 0.334) [117]. A similar OS benefit from ICI treatment, regardless of *BRAF* mutation status, was evidenced also in a sub-analysis of 11 patients with *BRAF*-mutant NSCLC enrolled in the Italian Expanded Access Program (EAP) for second-line nivolumab (median OS 11.2 months in the *BRAF* wild-type group and 10.3 months in those with *BRAF* mutations) [118]. In the subgroup with *BRAF* mutation enrolled in the IMMUNOTARGET registry (*n* = 43), an ORR of 24% and a PFS of 3.1 months were achieved [43]; however, PFS was positively associated with smoking status, confirming the key role of smoking history in response to immunotherapy. The improved outcomes observed with ICIs in *BRAF*-mutated NSCLC may be attributed to a higher prevalence of current or former smokers within this subgroup [119,120].

Furthermore, multiple studies have indicated that *BRAF*-mutated NSCLC is often associated with a high TMB and elevated PD-L1 expression [20]. Higher levels of TMB in *BRAF*-mutated patients compared to wild-type population (*p* = 0.009) were reported in the Zhang trial, but no significant differences in PD-L1 expression (*p* = 0.198) were noticed [117]. Conversely, a high PD-L1 expression (>50%) was detected in up to 50% of the 29 patients analyzed by Dudnik et al. (42% in the V600E cohort and 50% in the non-V600E cohort); additionally, 2 out of 11 patients presented high TMB [121]. Both PD-L1 expression and TMB are recognized predictive biomarkers for response to ICIs [20], explaining the possible benefit of immunotherapy in this population. However, some authors have suggested that this benefit is limited to patients with *BRAF* non-V600 mutations. In a retrospective analysis of 129 *BRAF*-mutated patients (*n* = 29 Class I, *n* = 59 Class II/III) ORR after ICI treatment was 9% in Class I-altered tumors and 26% in Class II/III (*p* = 0.25), reflecting the higher TMB evidenced in Class II/III mutations (8.8 mutations/Mb versus 4.9, *p*  <  0.001); notably, this difference was diminished when stratified by smoking status [122]. Although several other studies have investigated the outcomes of different BRAF mutation classes treated with ICIs, the evidence remains inconclusive [123].

Currently, despite the encouraging data discussed above, targeted therapies remain the up-front treatment strategy for patients with NSCLC and *BRAF* V600 mutations. Anti-PD-(L)1 monotherapy in patients with a PD-L1 TPS > 50%, as well as chemo-immunotherapy in those with lower PD-L1 expression, should be considered after the failure of BRAF/MEK inhibitors for V600 patients or as first-line treatment for non-V600 patients or for those considered unfit for TKIs, especially in cases of high PD-L1 expression or previous smoking history.

### 3.8. MET Alterations

Alterations in the *MET* gene, predominantly characterized by exon 14 skipping mutations and amplifications, occur in approximately 3–4% of NSCLC cases [124]. Historically, these tumors have demonstrated significant resistance to chemotherapy; however, the introduction of MET-targeted TKIs such as capmatinib and tepotinib has substantially reshaped the therapeutic paradigm for this subgroup [125,126,127]. From a molecular standpoint, MET alterations are now classified into two distinct categories: the exon 14 skipping subgroup and the MET amplification subgroups. The exon 14 skipping group, akin to other oncogene-driven mutations, is generally associated with low TMB, though emerging evidence suggests that some cases may exhibit elevated PD-L1 expression [128]. Meanwhile, the MET amplification subgroup can be further stratified into high and low amplification levels, with high amplifications often co-occurring with a multitude of other mutations, as observed in over 80% of cases in a retrospective analysis of 337 tumor specimens [129], and higher TMB levels.

Despite the scarcity of data on the efficacy of ICIs in MET-altered NSCLC, the available evidence presents conflicting results. In a retrospective analysis by Sabari et al. which included 147 patients with MET exon 14-altered NSCLC, the ORR to ICIs was reported at 17%, with a median PFS of 1.9 months [130]. Similarly, other retrospective analyses have indicated modest clinical activity, with the median PFS ranging between 2.7 and 3.4 months [20,43]. Conversely, a multicenter retrospective analysis conducted by Guisier et al. (*n* = 30) reported a more favorable ORR of 35.7% and a median PFS of 4.9 months, while data from the IMMUNOTARGET registry indicated a median PFS of 3.4 months and a median OS of 18.4 months, highlighting the potential variability in responses across different studies [43,76]. Further insight into the role of ICIs in MET-altered NSCLC comes from a case series of 24 patients with MET exon 14 skipping mutations and PD-L1 expression above 50%: these patients, treated with first-line pembrolizumab, achieved an ORR of 43%, a median PFS of 3.5 months, and an OS of 12.1 months, underscoring the potential utility of immunotherapy in select subpopulations [131]. A particularly noteworthy study by Kron et al. evaluated outcomes in 278 patients with MET amplifications, with a specific focus on those harboring a gene copy number (GCN) greater than 10. The analysis revealed a marked clinical benefit of immunotherapy in these patients, particularly when compared to chemotherapy. Patients with a GCN greater than 10 demonstrated a median OS of 36 months with ICIs versus 4 months with chemotherapy, while those with a GCN below 10 had a median OS of 19 months with immunotherapy compared to 8 months with chemotherapy. In contrast, no significant differences in response or survival were observed among patients with exon 14 skipping mutations when treated with immunotherapy versus chemotherapy [129].

Recently, a retrospective analysis by Blasi et al. reported outcomes of 110 patients with NSCLC harboring MET exon 14 skipping alterations [132]. Compared to chemotherapy alone, the combination of chemotherapy and immunotherapy was associated with a longer median PFS (6 vs. 2.5 months, *p* = 0.004), a higher, albeit not statistically significant, ORR (49% vs. 28%, *p* = 0.086), and a trend toward extended OS (16 vs. 10 months, *p* = 0.240). In patients receiving mono-immunotherapy, the OS (14 vs. 16 months, respectively) and duration of response (26 vs. 22 months, respectively) were comparable to those observed with the combination of chemotherapy and immunotherapy. Interestingly, tumors with TP53 mutations demonstrated a numerically higher ORR (56% vs. 32%, *p* = 0.088) and PFS (6 vs. 3 months, HR 0.67, *p* = 0.160), as well as significantly improved OS (21 vs. 11 months, HR = 0.54, *p* = 0.034), compared to TP53 wild-type tumors [132].

Collectively, these findings support the integration of selective MET TKIs into first-line treatment strategies for NSCLC harboring MET alterations, given the uncertain efficacy of standard chemo-immunotherapy in this subgroup [124]. Furthermore, these results underscore the critical need for precise molecular stratification in MET-altered tumors: by focusing on specific alterations, such as exon 14 skipping and GCN amplifications, alongside TP53 co-mutations, clinicians may enhance their ability to accurately predict which patients are most likely to derive substantial benefit from immunotherapy.

### 3.9. Perioperative Immunotherapy for NSCLC

The impact of ICIs has recently extended beyond the treatment landscape of locally advanced and metastatic NSCLC, marking a significant advance also in the perioperative setting. Several trials, including CheckMate 816 [133], PEARLS [134], and IMpower010 [135], demonstrated the efficacy of, respectively, preoperative nivolumab and adjuvant pembrolizumab or atezolizumab for operable NSCLC.

More recent efforts, however, have focused on a perioperative approach with protocols incorporating both neoadjuvant and adjuvant phases. The primary aim of this strategy is to harness the potential of neoadjuvant treatment to eradicate micrometastatic disease and minimize the extent of surgical resection while also leveraging the adjuvant phase to eradicate minimal residual disease in cases of suboptimal response. Within this evolving therapeutic framework, studies such as SAKK 16/14 [136], NADIM [137], NADIM II [137], AEGEAN [138], KEYNOTE-671 [139], TOP1501 [140], NeoTORCH [141], and CheckMate 77T [142] have reported meaningful improvements in pathological complete response (pCR), event-free survival (EFS), disease-free survival (DFS), and, in select instances, OS, establishing a new standard in the clinical management of these patients.

In this context, patients harboring *EGFR* or *ALK* mutations were generally considered ineligible for perioperative immunotherapy, with most major trials excluding this subgroup, while no data are available for the other AGAs, which have not been tested in any of the trials mentioned above. Although patients with *EGFR* mutations were included in the adjuvant trials IMpower010 and PEARLS, neither study was adequately powered to demonstrate benefits within this subset. However, interestingly, in the IMpower010 trial, where among patients with *EGFR*-positive tumors 53 were randomized to adjuvant atezolizumab and 64 to a placebo, subgroup analyses revealed comparable benefits across *EGFR*-positive, *EGFR*-negative, and unknown-status subgroups in the overall cohort, with HRs of 0.99, 0.79, and 0.70, respectively [135]. This trend was further corroborated in patients with PD-L1 expression ≥1%, where the corresponding HRs were 0.57, 0.67, and 0.61, respectively, underscoring a consistent therapeutic effect irrespective of *EGFR* mutation status [135].

A similar pattern emerged from the KEYNOTE-091 trial, wherein adjuvant pembrolizumab demonstrated a relatively greater benefit in patients harboring *EGFR* mutations (*n* = 39) compared to those with *EGFR* wild-type tumors and unknown status (HR= 0.44, HR = 0.78, and HR = 0.82, respectively) [143]. Furthermore, findings from the KEYNOTE-671 trial reinforced this observation, as perioperative pembrolizumab showed a more pronounced efficacy in *EGFR*-positive patients (*n* = 14, HR = 0.09) compared to those with *EGFR*-negative (HR = 0.48) or unknown-status tumors (HR = 0.64) [139]. However, these results must be interpreted with caution due to the limited sample size and statistical power of the subgroup analyses, emphasizing the necessity for powered clinical trials to draw definitive conclusions regarding the efficacy of ICIs in *EGFR*-mutated early NSCLC. Furthermore, the practice-changing findings from ADAURA [144] and ALINA [145] demonstrating the efficacy of adjuvant osimertinib in patients harboring *EGFR* mutations and adjuvant alectinib in patients harboring *ALK* mutations, alongside investigational perioperative strategies such as the neoADAURA [146] and NAUTIKA1 [147] approaches, highlight the prominent role of the targetable mutation also in early oncogene-addicted NSCLC.

### 3.10. New Therapeutic Strategies

**Figure 1:**
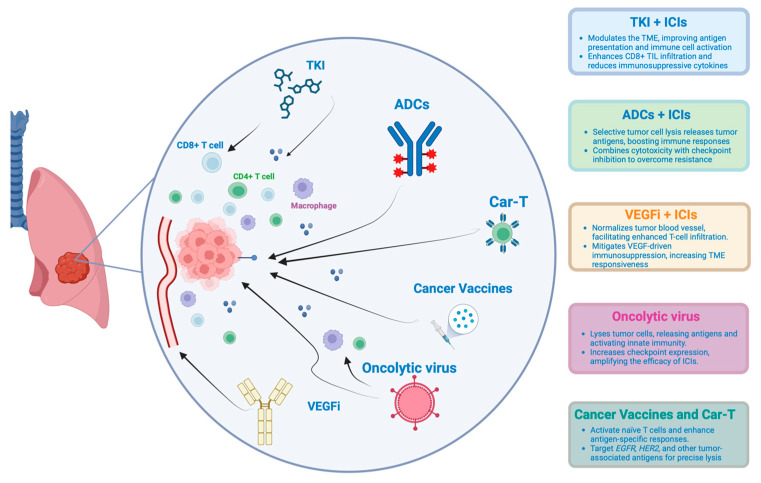
Emerging therapeutic strategies to enhance immunotherapy efficacy in oncogene-addicted NSCLC. This figure illustrates the interaction of various combination approaches with the tumor microenvironment (TME) in lung cancer. The approaches highlighted include tyrosine kinase inhibitors (TKIs), antibody–drug conjugates (ADCs), Vascular Endothelial Growth Factor Inhibitors (VEGFis), immune checkpoint inhibitors (ICIs), chimeric antigen receptor T cell therapy (CAR-T), cancer vaccines, and oncolytic viruses. Each therapeutic strategy is depicted in relation to its mechanism of action within the TME.

Given the variable efficacy of immunotherapy in NSCLC with AGA and the diverse immune evasion mechanisms used by tumor cells discussed above, new therapeutic strategies are currently under investigation (Figure 1). Integrating various immunotherapy approaches or combining them with other antitumor agents aims to activate immune responses against tumors, broaden the range of antitumor effects, and potentially overcome resistance mechanisms [148] (Table 2).

In subgroups like *KRAS* or *BRAF*, which have demonstrated higher response rates to ICIs treatment, combining TKI with immunotherapy could potentially enhance therapeutic synergy, modulating the tumor microenvironment. Preclinical data in *BRAF*-mutated cells showed that therapy targeting the MAPK pathway may induce immunological changes (increased CD4+ and CD8+ lymphocyte infiltrate, reduced release of immunosuppressive cytokines, and upregulated expression of the major histocompatibility complex I [149,150]), promoting a more favorable tumor microenvironment that may boost the tumor responses driven by ICIs. In this context, cohort E of the phase II/III B-FAST trial (NCT03178552) is designed to evaluate the efficacy and safety of a combination treatment with atezolizumab, vemurafenib, and cobimetinib in patients with advanced NSCLC harboring *BRAF* V600 mutations. Additionally, the antitumor activity of the combination of subcutaneous sasanlimab—a PD-1 antagonist monoclonal antibody—with encorafenib and binimetinib will be assessed in the phase Ib/II umbrella study Landscape 1011 (NCT04585815) [151].

Similarly, *KRAS* G12C inhibitors led to an enhanced influx and activation of CD8+ T cells, polarization of the myeloid compartment, increased antigen presentation, and upregulation of transcriptional programs associated with IFN signaling, changes that may boost the tumor responses driven by checkpoint blockade [152]. The phase Ib Codebreak 100/101 trial combining sotorasib and anti-PD-(L)1 antibodies (atezolizumab or pembrolizumab) showed promising efficacy (ORR 29%, DoR 17.9 months) but a greatly increased incidence of grade 3–4 liver toxicities [114]; conversely, the preliminary results from the combination of adagrasib and pembrolizumab in the phase II cohort of the KRYSTAL-7 (NCT04613596) trial did not result in substantial high-grade liver toxicities [153]. Phase III is ongoing to assess the efficacy of this strategy in the first-line setting in patients with *KRAS* G12C mutation and TPS ≥ 50%. New combinations of ICIs and more potent and selective *KRAS* G12C inhibitors are under investigation (GDC-6036 + atezolizumab in arm B of NCT04449874 trial and olomorasib + pembrolizumab +/− chemotherapy in SUNRAY-01 [NCT06119581]). Tislelizumab + trametinib (anti-MEK) + anlotinib (multikinase inhibitor) is also being tested in KRAS-mutant advanced NSCLC (NCT06456138).

Despite the limited effectiveness of immunotherapy in *ALK*-positive NSCLC, phase I/II studies evaluating the efficacy of *ALK* TKIs + ICI are ongoing (Table 2). In *EGFR* NSCLC, combining VEGF inhibitors with ICIs has emerged as a promising approach due to the potential of VEGF inhibitors to modulate the immune microenvironment, with preclinical and clinical evidence suggesting that they may promote T cell infiltration and immune activation [7,22]. In the phase III IMpower150 trial, the inclusion of anti-angiogenic agents, such as bevacizumab, alongside ICIs and chemotherapy was superior to either chemo-immunotherapy or chemo-anti-angiogenesis therapy on mPFS (10.2, 6.9, 6.9 months) and mOS (29.4, 19.0, 18.1 months) in a subgroup of *EGFR*-TKI-advanced *EGFR*-mutant NSCLCs [154]. Furthermore, the ORIENT-31 trial confirmed that the addition of a VEGF inhibitor to the combination of sintilimab and chemotherapy significantly improved clinical outcomes compared to chemotherapy alone in *EGFR*-mutated patients who had progressed following TKI therapy [21], confirming that VEGF inhibitors could potentially enhance the immunosensitivity of *EGFR*-mutated tumors and offer a viable therapeutic strategy for this subgroup of patients.

More recently, ADCs have been introduced as a new category of target therapy. Preliminary results of the TROPION-Lung02 (NCT04526691) trial showed a notable activity of the combination of datopotomab deruxtecan (Dato-DXd)—a TROP2-directed ADC—plus pembrolizumab with or without platinum chemotherapy in advanced pretreated NSCLC [155]; however, only patients with *KRAS* mutations were eligible for the study, while other AGAs were considered exclusion criteria. The TROPION-Lung04 trial aims to evaluate combination of Dato-DXd and immunotherapy +/− carboplatin in both treatment-naive and previously treated patients; cohorts 5–11 were recently added to evaluate Dato-DXd in combination with new bi-specific immunotherapies AZD2936 (rilvegostomig, anti-PD-1/TIGIT), MEDI5752 (volrustomig, anti-PD-1/CTLA-4) and AZD7789 (anti-PD-1/anti-TIM-3). Indeed, an increase in inhibitory checkpoint molecules, such as TIM-3, LAG-3, TIGIT, and BTLA, on CD8^+^ TILs and other cells in the TME could explain the various degrees of dysfunction of these cells involved in the pro-inflammatory response, including low proliferation, impaired cytokine production, and inability to lyse target cells [156].

Consequently, inhibiting these molecules through combinations of ICIs could represent a new therapeutic strategy to effectively impede tumor growth and promote the regression of cancer cells [157]. Of note, patients with AGAs are excluded from the TROPION-Lung04 trial. Conversely, the combination of volrustomig and rilvegostomig and T-DXd with or without chemotherapy will be specifically evaluated in patients with NSCLC and HER2 overexpression in the phase Ib DESTINY-Lung03 (NCT04686305) trial. The safety and efficacy of anti-PD-1 centrelimab combined with the bi-specific antibody amivantamb are also being tested in patients with NSCLC and driver mutations progressed after TT (phase I) or in patients with EGFR mutations (phase II) (NCT05908734).

Novel targets, such as the Cluster of Differentiation 39 (CD39)/Cluster of Differentiation 73 (CD73)/Adenosine A2A Receptor (A2AR) pathway, could also be considered. CD73, or ecto-5′-nucleotidase, is a cell surface enzyme expressed on various cell types, including tumor cells, and plays a critical role in modulating the tumor microenvironment [158]. By converting AMP into adenosine, CD73 fosters an immunosuppressive environment that suppresses antitumor immunity [159,160]. Adenosine inhibits the function of immune effector cells, such as T cells, NK cells, and macrophages while promoting the expansion and suppressive activity of regulatory T cells (Tregs) and myeloid-derived suppressor cells (MDSCs) [161]. Targeting CD73 has shown promise in preclinical models by restoring immune cell function and improving the efficacy of immune checkpoint inhibitors [162], making it a compelling target for combination therapies in immuno-oncology. CD73 expression is directly regulated by the Ras-Raf-ERK pathway, which is under the regulatory influence of *EGFR* signaling. *EGFR*-mutated NSCLC showed heightened CD73 expression compared to *EGFR* wild-type tumors and a combined administration of anti-PD-L1 and anti-CD73 antibodies significantly inhibited tumor growth, amplified the presence of tumor-infiltrating CD8^+^ T cells, and augmented the production of interferon-gamma (IFN-γ) and tumor necrosis factor-alpha (TNF-α) in this subgroup, emphasizing the rationale for combining anti-CD73 and anti-PD-L1 treatments [158]. Clinical trials of CD39 inhibitors are also under recruiting status currently.

Combining ICIs with cytokines might also enhance their efficacy. In vitro studies with *EGFR*-mutant human cell lines revealed that *EGFR*-mutant tumors with acquired resistance to *EGFR*-TKIs had an increased IL-6 secretion, leading to suppressed T- and NK-cell function. Blocking IL-6 enhances antitumor immunity and sensitizes *EGFR*-mutant tumors to PD-1 inhibitors [163]. Another study investigated the combination of anti-PD-1 with an adenovirus engineered to deliver tumor necrosis factor-alpha (TNF-α) and IL-2 in a mouse model of NSCLC: a reduction in cancer growth and an increased number of cytotoxic TILs were noticed, highlighting the potential of this approach [164].

Similarly, a combination of a PD-1 inhibitor and pegylated IL-10 demonstrated high antitumor activity in solid tumors, including NSCLC (ORR 43%) [165].

Given the high heterogeneity of cancer, oncolytic viruses (OVs) or therapeutic mRNA vaccines have also been evaluated to enhance ICIs’ efficacy. Oncolytic viruses specifically infect tumor cells and lyse them directly, stimulating the innate immune response through pathogen-associated and damage-associated molecular patterns, releasing tumor antigens and activating adaptive immune responses. Several studies have observed an increase in immune checkpoint expression following viral infection, leading to a more durable immune response when OVs are combined with ICIs [166]. Conversely, cancer vaccines can activate antigen presentation, generate tumor-specific T cells either in peripheral tissues or directly within the tumor, and promote their migration into the TME, increasing the presence of TILs. This strategy is currently being assessed in early-phase clinical trials in combination with pembrolizumab in patients with KRAS mutations. Notably, unlike ICIs, which boost the inactive responses of effector T cells, vaccination can activate tumor-specific naïve T cells, targeting the most common oncogenic mutations. More innovative approaches associate cancer vaccines to autologous T cells genetically engineered to express receptors reactive against a specific AGA (NCT06253520).

Moreover, T lymphocytes have been modified using synthetic chimeric antigen receptors (CARs) to target specific tumor associated antigens [167]. Anti-*EGFR* CAR-T cells exhibited specific cytolytic activity against *EGFR*-positive tumor cells in vitro and in mice [168]. Ongoing clinical trials are assessing the safety and efficacy of different approaches using modified anti-*EGFR* or anti-HER2 CAR-T cell therapy.

Despite all the advancements described above, the development of combination strategies continues to be an area of active exploration and further research is essential to determine the safety, efficacy, and long-term clinical benefits of these approaches in patients with oncogene-addicted NSCLC before their adoption in clinical practice.

In parallel, emerging evidence suggests that nutritional trace element supplementation, such as selenium and zinc, could play a supportive role in various cancers, including NSCLC management. Studies have highlighted the potential prognostic benefits of higher serum levels of selenium and zinc, which are associated with improved survival in lung cancer patients, potentially through mechanisms involving oxidative stress reduction and immune modulation [169]. Integrating such perspectives into multimodal treatment strategies may provide novel avenues to enhance the efficacy of immunotherapy while addressing systemic influences on tumor biology.

### 3.11. Conclusions

Emerging evidence highlights significant heterogeneity among NSCLC driver mutation subgroups treated with immunotherapy, suggesting that certain patients subsets may benefit from ICIs. In particular, *EGFR*- and *ALK*-altered tumors often present a “cold” immunophenotype, characterized by reduced TMB, relatively low PD-L1 expression, and immunosuppressive features—thus limiting immunotherapy benefit. By contrast, NSCLCs harboring *KRAS* or *BRAF* mutations, which frequently arise in smokers, typically exhibit higher TMB and an “inflamed” tumor microenvironment, resulting in comparatively stronger responses to ICIs, reflecting the significant influence of smoking status on immunotherapy outcomes and underscoring the intricate interplay between genetic alterations and environmental factors, such as smoking, in shaping the immune landscape of oncogene-driven NSCLC. *MET*- and *HER2*-driven disease, on the other hand, displays more heterogeneous immunogenic profiles, sometimes showing intermediate benefit from immunotherapy (e.g., *MET* exon 14 skipping). These subtype-specific differences underscore the clinical importance of refining biomarker strategies to further optimize patient selection and overcome immune resistance in oncogene-addicted NSCLC. In this context, combination therapies have emerged as a promising strategy to address immune resistance. The integration of ICIs with targeted therapies, VEGF inhibitors, ADCs, and bi-specific antibodies has shown potential to remodel the tumor microenvironment and enhance therapeutic efficacy. However, these approaches are tempered by the need to balance efficacy with the increased toxicity profiles associated with such regimens, necessitating careful patient selection and management. Although the underlying mechanisms of combination-therapy-related toxicity remain poorly understood, some evidence suggests that these toxicities may be drug-specific rather than class-specific, as observed with osimertinib combined with immunotherapy in the context of ILD risk compared to first- or second-generation TKIs [170]. Consequently, careful patient surveillance is crucial when implementing sequential therapy, requiring comprehensive clinical, laboratory, and radiological monitoring for the early detection of adverse events. In practice, this may involve routine assessments of thyroid and adrenal function to anticipate potential endocrine dysfunction, as well as regular imaging—such as computed tomography (CT) scans—to promptly identify early signs of ILD. Alongside these measures, an integrated pharmacological approach is essential to pinpoint potential drug–drug interactions and uncover toxicity-related biomarkers, ultimately allowing for timely intervention and dosage adjustments.

In parallel, the perioperative application of ICIs in oncogene-addicted NSCLC remains an area of significant challenge. Although early-stage tumors generally exhibit greater responsiveness to immunotherapy, most oncogene-driven subtypes—KRAS possibly being a notable exception—continue to display low immunogenicity, highlighting the difficulties in effectively integrating ICIs into the treatment of this molecularly distinct population. Identifying predictive biomarkers and refining molecular stratification may indeed enhance patient selection, maximizing therapeutic benefit while minimizing adverse effects. Continued efforts in this direction hold the potential to bridge the gap between molecular targeted therapy and immunotherapy, ultimately advancing personalized treatment approaches in the oncogene-addicted NSCLC subtypes.

## Figures and Tables

**Table 1:** Key prospective trials evaluating the efficacy of immunotherapy in oncogene-addicted NSCLC.

TRIAL	Phase	Treatment	ORR (%)	Median PFS (Months)	PMID
***EGFR***					
ATLANTIC (NCT02087423)	II	Durvalumab	11	1.9	29545095
NCT02879994	II	Pembrolizumab	0	4	29874546
OAK (NCT02008227)	II	Atezolizumab	NA	NA	27979383
BIRCH (NCT02031458)	II	Atezolizumab	Cohort 1: 23 Cohort 2: 0 Cohort 3: 7	Cohort 1: 5.5 Cohort 2: 1.3 Cohort 3: 1.4	28609226
CheckMate012 (NCT01454102)	Ib	Nivolumab + chemotherapy	Gemcitabine–cisplatin: 33 Pemetrexed–cisplatin: 47 Paclitaxel–carboplatin: 47	Gemcitabine–cisplatin: 5.7 Pemetrexed–cisplatin: 6.8 Paclitaxel–carboplatin: 4.8	27932067
NCT03924050	II	Toripalimab + chemotherapy	50	7.0	34650034
CheckMate 722 (NCT02864251)	III	Nivolumab + chemotherapy	31.3	5.6	38252907
KEYNOTE-789 (NCT03515837)	III	Pembrolizumab + chemotherapy	29	5.6	39173098
ORIENT-31 (NCT03802240)	III	Sintilimab + IBI305 + chemotherapy	44	6.9	37156249
TATTON (NCT02143466)	Ib	Osimertinib + durvalumab	Part A: 43 Part B: 82	Part A: NA Part B: 9.0	32139298
Impower150 (NCT02366143)	III	Atezolizumab + bevacizumab + chemotherapy	71	10	34311108
Checkmate 012 (NCT02864251)	Ib	Erlotinib + nivolumab	15	5	27932067
Ma15.02 (NCT02013219)	Ib	Erlotinib + atezolizumab	75	15	36871392
KEYNOTE-021 (NCT02039674)	I/II	Pembrolizumab + erlotinib/gefitinib	Erlotinib: 41.7 Gefitinib: 14	Erlotinib: 19 Gefitinib: 1	30529597
***KRAS***					
CheckMate 057 (NCT01642004)	III	Nivolumab vs. docetaxel	KRAS and TP53 co-mutations: 57 KRAS and STK11 co-mutations: 0 KRAS and KEAP1 co-mutations 18	NA	33449799
Impower150 (NCT02366143)	III	Atezolizumab + bevacizumab + chemotherapy	NA	8	34311108
CodeBreak100/101 (NCT03600883)	I	Sotorasib + pembrolizumab/atezolizumab	29	NA	//
***ALK***					
CheckMate 370 (NCT02393625)	I/II	Nivolumab + crizotinib	38	NA	29518553
JAVELIN Lung 101 (NCT02584634)	Ib	Avelumab + crizotinib or lorlatinib	46	NA	39034968
NCT02012219	Ib	Alectinib + atezolizumab	86	NA	35875467

**Table 2:** Main phase I/II clinical trials evaluating the combination of immunotherapy with ADCs or target therapies.

TRIAL	Phase	Target	Treatment
B-FAST (NCT03178552)	II/III	BRAF V600	Atezolizumab + vemurafenib + cobimetinib
Landscape 1011 (NCT04585815)	Ib/II	BRAF V600	Encorafenib + binimetinib + sasanlimab
SUNRAY-01 (NCT06119581)	1/2	KRAS G12C	LY3537982 + pembrolizumab
NCT044498v74	II	KRAS G12C	GDC-6036 + atezolizumab
NCT06456138	I/II	KRAS G12C	Tislelizumab + trametinib + anlotinib
DESTINY-Lung03 (NCT04686305)	Ib	HER2	T-DXD with durvalumab with or without chemotherapy
HUDSON (NCT03334617)	II Umbrella	HER2	T-DXD with durvalumab
TROPION-Lung02 (NCT04526691)	Ib	TROP2	Datopotamab deruxtecan plus permbrolizumab with or without chemotherapy
TROPION-Lung04 (NCT04612751)	Ib	TROP2	Datopotamab deruxtecan plus immunotherapy with or without chemotherapy
NCT04306900	I/Ib	CD39	TTX-030 + immunotherapy
NCT06507306	I/1b	SOS1	KQB198 + osimertinib
NCT05067283	I	KRAS G12C	K-1084 + pembrolizumab
TACTI-004	III	LAG-3	Eftilagimod alfa plus pembrolizumab

## Data Availability

Data sharing is not applicable to this article as no new data were created or analyzed in this study.
